# The Prevalence of Adenoid Hypertrophy among Children with Zika Related Microcephaly

**DOI:** 10.3390/v13010062

**Published:** 2021-01-05

**Authors:** Mariana C. Leal, Danielle Seabra Ramos, Thiago Pinto Bezerra, Ana Elizabeth S. C. Vilela, Rebeka Jacques de F. Maciel, Mirella Rodrigues, Mariana Lira, Karen Pena de Souza Cavalcanti, Vanessa Van der Linden, Marli T. Cordeiro, Demócrito Miranda-Filho, Ricardo Ximenes, Elizabeth B. Brickley, Silvio S. Caldas

**Affiliations:** 1 Surgery Department, Federal University of Pernambuco, Recife 50670-901, Brazil; danielle.seabra@unicap.br (D.S.R.); pintobezerra@gmail.com (T.P.B.); rebekajacques@gmail.com (R.J.d.F.M.); mirellarod@hotmail.com (M.R.); raaximenes@uol.com.br (R.X.); silvio_caldas@oi.com.br (S.S.C.); 2 Agamenon Magalhães Hospital, Recife 52070-230, Brazil; ana_beth18@hotmail.com; 3 Medicine Department, Catholic University of Pernambuco, Recife 50050-900, Brazil; 4 Clinical Hospital, Federal University of Pernambuco, Recife 50740-900, Brazil; marianammlira@yahoo.com.br (M.L.); karenkpsc@yahoo.com.br (K.P.d.S.C.); 5 Immunopathology Laboratory Keizo Asami, Federal University of Pernambuco, Recife 50670-901, Brazil; 6 Barão de Lucena Hospital, Recife 50731-000, Brazil; vanessavdlinden@hotmail.com (V.V.d.L.); marli.tenorio@gmail.com (M.T.C.); 7 Oswaldo Cruz University Hospital, University of Pernambuco, Recife 50100-130, Brazil; demofilho@gmail.com; 8 London School of Hygiene & Tropical Medicine, London WC1E 7HT, UK; elizabeth.brickley@lshtm.ac.uk

**Keywords:** Zika, microcephaly, adenoids, nasal obstruction, dysphagia, secretory otitis media

## Abstract

Upper respiratory obstruction is a common sequela in children with Zika-related microcephaly (ZRM). As a cross-sectional analysis nested in a cohort study, this study aims to investigate the prevalence of adenoid hypertrophy (AH) in children with ZRM and symptoms of respiratory obstruction. The data were collected in the first three years of life from children with ZRM who were followed in two reference centers for otorhinolaryngological care of patients with congenital Zika syndrome. Out of 92 children with confirmed ZRM, 57 were evaluated by nasopharyngoscopy after presenting with upper respiratory obstruction symptoms. In this study, 31 of the 57 (54%) children with ZRM who were evaluated had obstructive AH. Thirteen children with obstructive AH were submitted to surgery, which resulted in the complete resolution of symptoms for 11, partial resolution in 1, and no improvement in 1. No evidence of direct involvement by Zika virus (ZIKV) infection in the adenoid tissues was demonstrated by histology or immunohistochemistry. Our results suggest that there is a high prevalence and early presentation of AH in children with ZRM, with consequent upper airway obstruction causing upper airway obstructive disorder, secretory otitis media, and dysphagia.

## 1. Introduction

In late 2015, Brazil experienced a dramatic increase in microcephaly cases, which were subsequently attributed to intrauterine infection by Zika virus (ZIKV) [1,2,3,4,5,6] Much has been written about congenital ZIKV infection since then; however, the full spectrum of congenital Zika syndrome (CZS) is not yet known. Microcephaly is the most severe and distinctive manifestation of CZS and is often associated with cerebral cortex hypoplasia and subcortical calcifications. Macular scars and focal retinal pigmentation, hypertonia, extrapyramidal symptoms, and dysphagia are other findings and symptoms that are now recognized to be related to CZS [7,8,9,10,11,12]. Follow-up studies in children with and without microcephaly are essential for understanding the sequelae of CZS over the early life course. As part of a prospective cohort of children with CZS in Recife, Pernambuco, Brazil, this study aims to investigate adenoid hypertrophy (AH) as a cause of upper respiratory obstruction in children with Zika-related microcephaly (ZRM) in the first three years of life.

Resulting from alterations in the normal growth of lymphoid tissue, hypertrophy of the pharyngeal tonsils, better known as adenoids, can present with a range of symptoms including nasal obstruction, mouth breathing, craniofacial alterations, dysphagia, conductive hearing losses related to secretory otitis media, and snoring during sleep. In more severe cases, AH may result in obstructive sleep apnea (OSA) with severe systemic consequences, such as cardiovascular morbidity, metabolic disorders, and nocturnal enuresis [13,14,15,16,17]. Although not fully understood, the pathophysiology of OSA in childhood is thought to have a very close relationship with lymphoid tissue hypertrophy in the upper airway, especially in the pharyngeal and palatine tonsils [13]. 

Few studies have evaluated adenoid growth in the general population. Papaioannou et al., in a magnetic resonance imaging study, evaluated the diameter of the nasopharynx and the size of the adenoid in children aged 0 to 15 years and reported that the transversal diameter of the nasopharynx did not grow between 0 and 4 years, grew slowly between 4 and 8 years, and then presented rapid growth from age 8 onwards for children without complaints of sleep-associated breathing disorders. In contrast, children with snoring appeared to present with progressive linear growth of the adenoid over the first 15 years, which was associated with a reduced growth of the nasopharynx cross-sectional area [17]. Consistent with these observations, population studies describing the profile of adenoidectomies carried out on the European and Asian continents over the last decade show that the performance of this type of surgery peaks between 3 and 5 years of age and takes place rarely below 2 years [18,19]. 

Of relevance to CZS, children with neurological diseases are at increased risk for sleep-associated breathing disorders due to a number of factors that include reduced muscle tone, especially weakness of respiratory muscles, and the prevalence of epilepsy. Additional factors such as macroglossia, obesity, and AH can worsen sleep-associated breathing disorders and lead to a more severe pattern of OSA with long-term systemic repercussions [20,21]. In children with ZRM, it has been hypothesized that upper airway obstruction from AH has the potential to further aggravate limitations resulting from the children’s CZS-associated neurodevelopmental delays. To better understand the role of AH in obstructive respiratory symptoms in children with ZRM, this study evaluates clinical data collected in the first three years of life from children with ZRM who were followed in two reference centers for otorhinolaryngological care of patients with CZS. 

## 2. Methods

We conducted a cross-sectional study nested in a cohort. Between September 2016 and July 2018, we evaluated 92 children with ZRM referred for tertiary care in otorhinolaryngology to the Hospital Agamenon Magalhães and Hospital das Clínicas (Recife, Pernambuco, Brazil). The Research Ethics Committee of Oswaldo Cruz University Hospital (CAAE: 52803316.8.0000.5192) provided ethical approval for this study on 10 October 2016.

The primary aim of the cross-sectional study was to describe upper airway abnormalities, including the prevalence of AH, in children with ZRM. The study sample included children with congenital ZIKV infection confirmed through laboratory testing of cerebrospinal fluid by a positive Zika virus-specific immunoglobulin M (IgM) capture enzyme-linked immunosorbent assay (ELISA) performed on cerebrospinal fluid (conducted in accordance with the CDC protocol) [18,19]. For differential diagnosis to exclude other potential causes of microcephaly in the children, serologic tests were performed on both mother and newborns for cytomegalovirus, toxoplasmosis, syphilis, and HIV. When cytomegalovirus IgG was present in both mother and child, further PCR testing for cytomegalovirus DNA was performed in urine or blood.

We used a standard form to collect demographic and clinical data from the maternal-child dyads related to pregnancy (self-reported presence and timing of rash during pregnancy), birth (gestational age, birthweight, and head circumference at birth), and some postnatal data (head circumference at follow-up) [22].

All described investigations were conducted as part of the clinical protocol or for a clinical indication; none were conducted for research reasons alone. In this study, microcephaly was defined as a head circumference (also known as occipitofrontal circumference) of more than two standard deviations below the mean for gestational age and sex. The degree of microcephaly was evaluated, and severe microcephaly was defined as head circumference at birth of at least three standard deviations below the mean for gestational age and sex [23]. Microcephaly was defined, at birth, using the standards of the Fetal International and Newborn Growth Consortium Description and Prevention for the 21st Century (INTERGROWTH-21st; https://intergrowth21.tghn.org/) and, in infancy, the World Health Organization (WHO) Child Growth Standards (www.who.int/childgrowth/en/). All children included in this study had microcephaly at the time of the otorhinolaryngological examination, but five were born with normal head circumferences and subsequently developed microcephaly according to the WHO Child Growth Standards in the first months of life. Birth weight was also evaluated using INTERGROWTH-21st standards and classified as appropriate, small, or large for gestational age and sex. 

After obtaining informed consent by the parents or guardians, we conducted complete otorhinolaryngological examinations for all of the children and collected, through interviews with parents or caregivers during the medical consultations, clinical data about history of obstructive respiratory symptoms and dysphagia. In the subset of children with reported nasal obstruction, snoring, mouth breathing, apnea, or swallowing disorders, we performed flexible nasopharyngoscopy using a 2.7 mm diameter flexible endoscope coupled to an LED light source and a high-definition camera by Karl Storz^®^, Walsdorf, Germany.

All exams were recorded on a hard drive. Nasopharyngoscopies were performed after preparing the nose with lidocaine spray in both sides. All exams were undertaken by two of the authors (Mariana C. Leal and Thiago Pinto Bezerra). Nasopharyngoscopy has been widely used for AH diagnosis, with a sensitivity of 92% and specificity of 71% [24].

The criteria for grading AH by nasopharyngoscopy vary across the published literature, and both objective and subjective measures are used. In this study, adenoid size was graded endoscopically according to the methods of Parikh et al. [25] into four grades: grade 1 indicates no contact with adjacent structures (i.e., physiological condition); grade 2 indicates contact with the torus tubarius; grade 3 indicates contact with the vomer; and grade 4 indicates contact with the soft palate. For this study, we considered grades 1 and 2, which have at least 50% of the nasopharyngeal cross-sectional area available for airflow, as non-obstructive, and grades 3 and 4 as obstructive or hypertrophic, with obstruction of greater than 50% of the nasopharyngeal cross-sectional area. Grades 3 and 4 were used to identify patients who are candidates for surgery [25,26].

Otoscopy was performed to evaluate abnormalities of the middle ear potentially related to AH (signs of Eustachian tube dysfunction, e.g., tympanic retractions or middle ear effusion). The oropharynx was also evaluated to identify palatine tonsils, using Brodsky grading scale [22], as follows: (0) tonsils are entirely within the tonsillar fossa; (1+) tonsils are visible but occupy less than 25% of the lateral dimension of the oropharynx as measured between the anterior tonsillar pillars; (2+) tonsils occupy less than 50 percent of the lateral dimension of the oropharynx; (3+) tonsils occupy less than 75 percent of the lateral dimension of the oropharynx; and (4+) tonsils occupy more than 75% of the lateral dimension of the oropharynx. We considered 3+ or 4+ as palatine tonsils hypertrophy.

Patients with respiratory obstruction related to adenoid/tonsil hypertrophy underwent surgery, and tissues were collected for histopathological study. The specimens were fixed in 10% buffered formaldehyde, processed, and stained in hematoxylin-eosin for histological analysis under light microscopy. In addition, tissue specimens were prepared for immunohistochemical analysis using a polymer-based indirect colorimetric immunoalkaline phosphatase detection system (MACH 4™ Universal AP Polymer Kit, MRA536L, by BIOCARE medical®, Pacheco, CA, USA) with fast red chromogen (Lab Vision™ Fast-Red Substrate System, TA-125-AF, by Thermo Scientific™, Waltham, MA, USA) according to the protocol of Martinez et al. (2016), adapted for the manual procedure. In this assay, we used a mouse monoclonal anti-Zika virus antibody (ARG65781 anti-Zika virus NS1 antibody [SQab1609] by Zika Arigo Laboratories® Gongyuan Road Hsinchu City 300 Taiwan, ROC) at a dilution of 1:500. Vero E6 kidney cells that had been inoculated with Zika virus and harvested, fixed in formalin, and embedded in paraffin were used as a positive control. Negative controls were also run in parallel and consisted of sequential tissue sections from case patients each incubated with normal mouse serum [27].

Data were expressed in absolute and relative frequencies. Likelihood ratio tests (for 3 × 2 tables) and Fisher’s exact tests (for 2 × 2 tables) were used to investigate the association between categorical variables. All analyses were performed in SPSS Statistics Rel. 20.0.0 (SPSS, Chicago, IL, USA), and the level of significance was set at *p* < 0.05.

## 3. Results

Of the 92 children who underwent a complete otorhinolaryngological examination in this study, 57 (62.0%) presented with symptoms of upper airway obstruction and were submitted to nasopharyngoscopy. The mean age of this subgroup was 22.9 months (range: 9 to 34 months); 27 (47.4%) were female and 30 (52.6%) were male. Forty-five (78.9%) mothers reported a rash during pregnancy and 70% (*N* = 31/44) of the mothers with known timing of rash during pregnancy were affected in the first trimester of pregnancy. The majority of infants were born full-term (89.5%, *N* = 51/57) and with appropriate weight for gestational age (73%, *N* = 42/57) (Table 1).

Nasopharyngoscopy revealed obstructive AH (grades 3 or 4) in 54.4% of the children (*N* = 31/57), while palatine tonsil hypertrophy was present in only 3 (5.3%) patients, all of whom had associated AH. Secretory otitis media or otitis media with effusion (OME) was diagnosed in 24.6% (*N* = 14/57) of the children, and 13 of the 14 (92.9%) children with OME had AH. Of the obstructive respiratory symptoms, the most frequent complaints were snoring and nasal obstruction, which presented respectively in 64.9% (*N* = 37/57) and 62.5% (*N* = 35/56) of the children evaluated, while 25% (*N* = 13/52) had at least one episode suggestive of sleep apnea. The prevalence of these symptoms was associated with endoscopic AH findings. Among the children with AH, snoring and nasal obstruction were reported in at least 90%, while apnea was reported in 39% (*N* = 11/28) (Table 2).

Thirteen of the children who had symptomatic grade 3 or 4 AH underwent surgical treatment. Eight (61.5%) children underwent adenoidectomy and five (38.5%) children underwent adenoidectomy and myringotomy with ventilation tube placement for OME. Overall, 11 children had complete resolution of respiratory symptoms, 1 child had partial resolution, and 1 child did not have any improvement. Adenoidectomy also resulted in considerable improvement in dysphagia for 10 of the 12 (83.3%) patients who had dysphagia before surgery; notably, only the 2 children who had severe dysphagia prior to surgery did not improve as a result of surgery. 

**Figure 1:**
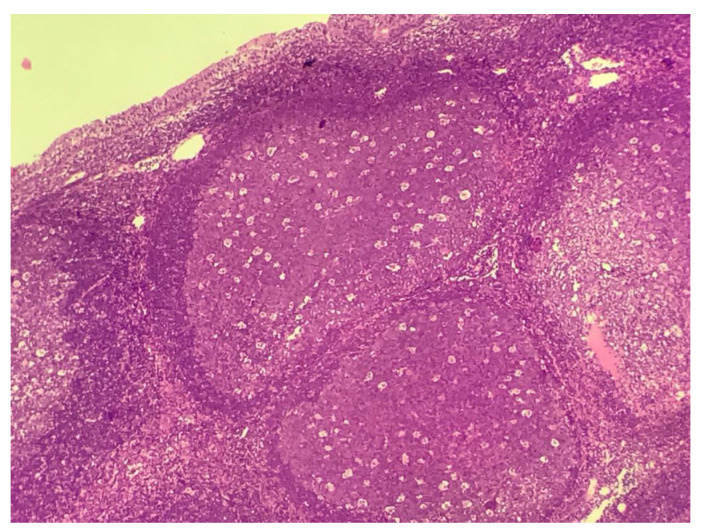
Bulky lymphoid follicles with activated germinal centers (hematoxicillin-eosin, 10×).

In the histological analysis, all samples consisted of bulky lymphoid follicles with activated germinal centers, indicating reactive hyperplasia. No multinucleated giant cells or cytopathic changes that could be attributed to viral action were identified (Figure 1). In immunohistochemical analysis, no ZIKV NS1 antigen was identified. 

## 4. Discussion

We observed obstructive AH in more than half of children with ZRM in this study. These data reveal the importance of evaluating the upper airway obstruction symptoms in children with ZRM, even in the first years of life. Despite the absence of AH prevalence studies in the population under 3 years old, the average age of our sample was 22.9 months. Therefore, we consider the 54% of AH observed in our sample a high and relevant prevalence, especially compared with data from surgical studies that show a peak performance of adenoidectomy between 3 and 5 years and rarely before the second year of life. Even in the casuistry of our service in the 2018–2020 biennium, among the 135 children (without microcephaly) who underwent adenoidectomy or adenotonsillectomy, only three were younger than 3 years of age.

The current study is also the first investigation to evaluate respiratory complaints, such as nasal obstruction, snoring, and apnea, in children with ZRM, and these symptoms were found to be very prevalent (62.5% (35/56), 64.9% (37/57), and 25% (13/52) respectively) in the study population. 

The adenoid is a secondary lymphoid tissue of the mucosa-associated lymphoid tissue (MALT). At birth, there are no lymphoid follicles in the adenoid tissue, but this tissue gradually develops with exposure to environmental antigens. The greatest increase in volume of the palatine tonsils occurs from 4 to 10 years of age, while the volume of the pharynx increases the most from 3 to 7 years. This physiological and natural hyperplasia accompanies the child’s lymphoid maturation process, and there is a physiological involution of these tissues after puberty. When this tissue reaches its peak of growth, nasal breathing and swallowing should occur naturally, without clinical symptoms. Therefore, AH with obstructive symptoms in the first years of life is very unlikely to occur. [28,29,30]

Our study found a prevalence of AH of greater than 50% among infants with ZRM. Although this prevalence of AH is substantial, studies of the prevalence of AH in the general population are scarce, especially in children younger than 3 years. In a recent meta-analysis about AH prevalence, the mean age of the participants in all of the studies included was higher than 5 years. However, the authors affirmed that it was not possible to specify prevalence in age groups due to heterogeneity in patients’ age between the included studies [17,18,19,30]. 

Although there are few studies that define the association between a specific viral infection and adenoid lymphoid tissue hypertrophy, viral genetic material, such as DNA from the herpesviridae family that include herpes simplex viruses, cytomegalovirus, and Epstein-Barr virus, has been detected in adenoid samples of children undergoing adenoidectomy [31]. In another study with HIV-positive patients, the most common and early rhinologic manifestation was AH, with a prevalence of 41.6% suggesting that viral infections may play a role in the immunopathogenesis of AH [32]. For this reason, investigating a possible direct effect of ZIKV infection or a specific reaction to it in adenoid tissue was an important consideration, although neither histological nor immunohistochemical studies demonstrated any finding that could support this hypothesis.

One factor that may be related to a higher frequency of AH in the studied children is the craniocephalic disproportion that characterizes the severe cases of ZRM. This feature might promote a relative increase of the adenoid in a disproportionately smaller rhinopharynx, which could contribute to the early onset of obstructive symptoms. The high frequency of early-age AH in ZRM deserves special attention and early intervention. The clinical repercussions secondary to nasal obstruction can lead to worsening dysphagia in addition to disordered sleep behavior that is related to behavioral changes such as attention deficits, in addition to the impact on craniofacial growth [26].

In this study, almost all of the children who were surgically treated for AH had full resolution of their upper airway obstructive symptoms, without any report of intra- or post-operative complications. One child who had only a slight improvement and continues to require a continuous positive airway pressure (CPAP) machine because of severe apnea is still being investigated. Dysphagia symptoms also improved, according to the children’s caregivers’ reports. Dysphagia has been one of the most frequent symptoms in patients with ZRM and may have an impact on the morbidity and mortality of these children [33]. Therefore, the otorhinolaryngological evaluation of children with ZRM is important and should include evaluations related to the anatomy and functioning of the upper aero-digestive tract for the identification and early treatment of disorders such as AH.

This study provides evidence that surgical treatment may yield therapeutic benefits for respiratory and digestive function as well as the hearing system and may consequently improve the quality of life for children with ZRM and AH.

### Limitations

An important limitation of the study is that, for ethical reasons, only children with symptoms consistent with upper airway obstruction underwent nasopharyngoscopy. Therefore, it was not possible to estimate the prevalence of AH in the whole population of patients with ZRM. However, among the children evaluated by nasofibroscopy, more than half were diagnosed with AH, and the presence of obstructive AH was significantly associated with the presence of snoring, nasal obstruction, and apnea. These findings provide evidence that symptomatic AH presents at an early age in children with ZRM.

A second limitation is that the sample of children with ZRM evaluated in this study represents the most severe spectrum of CZS cases. Indeed, our data indicate that approximately 70% of children with AH had severe microcephaly. Subsequent studies involving a wider spectrum of children with CZS would allow better clarification of the association between congenital ZIKV infection and AH.

Several questions about the immunopathology of AH remain unanswered. Further studies investigating adenoid tissue immunology in children with CZS will be important for defining the pathogenesis of AH in this population. In addition, upper airway imaging assessments during the period of craniofacial development of children with ZRM will be valuable for comparisons with the general population, which could help anticipate the need for surgical intervention in children with ZRM and AH. 

## 5. Conclusions

Our results suggest that there is a high prevalence and early presentation of AH in children with ZRM that may lead to upper airway obstructive disorder, secretory otitis media, and dysphagia. Monitoring these children during the first years of life is important to ensure the early recognition of upper airway obstructive disorder and timely referral to appropriate therapy for reducing complications from adenoid hypertrophy.

## Figures and Tables

**Table 1:** Selected characteristics by adenoid hypertrophy (AH) status of infants with Zika-related microcephaly and symptoms of upper respiratory obstruction (*N* = 57).

Characteristics		AH Classification		Total.	*p*-Value
		Obstructive AH	Non-Obstructive AH		
		*n* (%)	*n* (%)	*n* (%)	
Sex		*n* = 31	*n* = 26	*n* = 57	
	Female	14 (45.2)	13 (50.0)	27 (47.4)	0.461^1^
	Male	17 (54.8)	13 (50.0)	30 (52.6)	
Gestational age at birth		*n* = 31	*n* = 26	*n* = 57	
	Preterm	2 (6.5)	4 (15.4)	6 (10.5)	0.254 ^1^
	Term	29 (93.5)	22 (84.6)	51 (89.5)	
Weight for gestational age at birth		*n* = 31	*n* = 26	*n* = 57	
	SGA	6 (19.4)	6 (23.1)	12 (21.1)	0.684 ^2^
	AGA	24 (77.4)	18 (69.2)	42 (73.7)	
	LGA	1 (3.2)	2 (7.7)	3 (5.3)	
Self-reported rash during pregnancy		*n* = 31	*n* = 26	*n* = 57	
	Yes	25 (80.6)	20 (76.9)	45 (78.9)	0.491 ^1^
	No	6 (19.4)	6 (23.1)	12 (21.1)	
Timing of rash during pregnancy		*n* = 24	*n* = 20	*n* = 44	
First trimester		17 (70.8)	14 (70.0)	31 (70.5)	0.439 ^2^
Second trimester		7 (29.2)	5 (25.0)	12 (27.3)	
Third trimester		0 (0.0)	1 (5.0)	1 (2.3)	
Degree of microcephaly		*n* = 28	*n* = 25	*n* = 53	
Severe microcephaly (>3 SD below mean)		17 (60.7)	21 (84.0)	38 (71.7)	0.074 ^1^
Moderate microcephaly (>2 to 3 SD below mean)		11 (39.3)	4 (16.0)	15 (28.3)	

SGA: small for gestational age; AGA: appropriate for gestational age; LGA: large for gestational age.^1^ Fisher’s exact test; ^2^ Likelihood ratio test.

**Table 2:** Selected symptoms by AH status of infants with Zika-related microcephaly (*N* = 57).

Symptoms and Characteristics		AH Classification		Total	*p*-Value
		Obstructive AH	Non-Obstructive AH		
		*n* (%)	*n* (%)	*n* (%)	
Sleep apnea		*n* = 28	*n* = 24	*n* = 52	
	Yes	11 (39.3)	2 (8.3)	13 (25.0)	0.011 ^1^
	No	17 (60.7)	22 (91.7)	39 (75.0)	
Snoring		*n* = 31	*n* = 26	*n* = 57	
	Yes	28 (90.3)	9 (34.6)	37 (64.9)	<0.001 ^1^
	No	3 (9.7)	17 (65.4)	20 (35.1)	
Nasal obstruction		*n* = 30	*n* = 26	*n* = 56	
	Yes	27 (90.0)	8 (30.8)	35 (62.5)	<0.001 ^1^
	No	3 (10.0)	18 (69.2)	21 (37.5)	
Dysphagia		*n* = 30	*n* = 24	*n* = 54	
	Yes	27 (90)	22(91.6)	49 (90.7)	1 ^1^
	No	3 (10)	2(8.3)	5 (9.3)	
Palatine tonsils hypertrophy		*n* = 31	*n* = 26	*n* = 57	
	Yes	3 (9.7)	0 (0.0)	3 (5.3)	0.154 ^1^
	No	28 (90.3)	26 (100.0)	54 (94.7)	
Middle ear effusion		*n* = 31	*n* = 24	*n* = 55	
	Uni	4 (12.9)	0 (0.0)	4 (7.3)	0.002 ^2^
	Bi	9 (29.0)	1 (4.2)	10 (18.2)	
	No	18 (58.1)	23 (95.8)	41 (74.5)	

^1^ Fisher’s exact test; ^2^ Likelihood ratio test.

## Data Availability

The data presented in this study are available in : Leal, M.C.; Seabra Ramos, D.; Pinto Bezerra, T.; Vilela, A.E.S.C.; Maciel, R.J.d.F.; Rodrigues, M.; Lira, M.; Cavalcanti, K.P.d.S.; Van der Linden, V.; Cordeiro, M.T.; et al. The Prevalence of Adenoid Hypertrophy among Children with Zika Related Microcephaly. *Viruses* **2021**, *13*, 62. https://doi.org/10.3390/v13010062.
